# Resilience and phenotypic plasticity of Arctic char (*Salvelinus alpinus*) facing cyclic hypoxia: insights into growth, energy stores and hepatic metabolism

**DOI:** 10.1093/conphys/coad099

**Published:** 2023-12-15

**Authors:** Loïck Ducros, Mohamed Touaibia, Nicolas Pichaud, Simon G Lamarre

**Affiliations:** Département de Biologie, Université de Moncton, 18 Antonine Maillet, Moncton E1A 3E9, NB, Canada; Département de Chimie et Biochimie, Université de Moncton, 18 Antonine Maillet, Moncton E1A 3E9, NB, Canada; Département de Chimie et Biochimie, Université de Moncton, 18 Antonine Maillet, Moncton E1A 3E9, NB, Canada; Département de Chimie et Biochimie, Université de Moncton, 18 Antonine Maillet, Moncton E1A 3E9, NB, Canada; Département de Biologie, Université de Moncton, 18 Antonine Maillet, Moncton E1A 3E9, NB, Canada

**Keywords:** acclimation, fish, growth, hypoxia, metabolites, reoxygenation, resilience

## Abstract

Arctic char (*Salvelinus alpinus*) is facing the decline of its southernmost populations due to several factors including rising temperatures and eutrophication. These conditions are also conducive to episodes of cyclic hypoxia, another possible threat to this species. In fact, lack of oxygen and reoxygenation can both have serious consequences on fish as a result of altered ATP balance and an elevated risk of oxidative burst. Thus, fish must adjust their phenotype to survive and equilibrate their energetic budget. However, their energy allocation strategy could imply a reduction in growth which could be deleterious for their fitness. Although the impact of cyclic hypoxia is a major issue for ecosystems and fisheries worldwide, our knowledge on how salmonid deal with high oxygen fluctuations remains limited. Our objective was to characterize the effects of cyclic hypoxia on growth and metabolism in Arctic char. We monitored growth parameters (specific growth rate, condition factor), hepatosomatic and visceral indexes, relative heart mass and hematocrit of Arctic char exposed to 30 days of cyclic hypoxia. We also measured the hepatic protein synthesis rate, hepatic triglycerides as well as muscle glucose, glycogen and lactate and quantified hepatic metabolites during this treatment. The first days of cyclic hypoxia slightly reduce growth performance with a downward trend in specific growth rate in mass and condition factor variation compared to the control group. This acute exposure also induced a profound metabolome reorganization in the liver with an alteration of amino acid, carbohydrate and lipid metabolisms. However, fish rebalanced their metabolic activities and successfully maintained their growth and energetic reserves after 1 month of cyclic hypoxia. These results demonstrate the impressive ability of Arctic char to cope with its changing environment but also highlight a certain vulnerability of this species during the first days of a cyclic hypoxia event.

## Teaser

Arctic char, a cold-water fish, faces challenges like rising temperatures and low oxygen levels in its southernmost habitats. We studied how they adapt to cyclic low oxygen (hypoxia) events. Initially, hypoxia appears to reduced growth, affected metabolism, and altered liver functions. However, over time, Arctic char adjusted and maintained their growth and energy stores. While they can adapt, they are vulnerable during the initial stages of hypoxia.

## Introduction

The Arctic char (*Salvelinus alpinus*) is known for being the northernmost freshwater fish species in the world. This salmonid is indeed primarily distributed in Arctic and subarctic coastal and inland waters. However, it can also be found further south in cool waters (Klemetsen *et al.,* 2003). Arctic char occupies the upper part of the trophic chain and is the only apex predator in several communities (Klobucar and Budy, 2020). This fish represents an important food and financial source for many Inuit communities (Harris *et al.,* 2023). Arctic char is also a cultural and economic resource for several countries in Europe (Eriksson *et al.,* 2006; Caudron *et al.,* 2014; Winfield *et al.,* 2019). However, several southern populations are in decline or have even disappeared in recent years, in part due to climate warming and eutrophication (Igoe *et al.,* 2003; Maitland *et al.,* 2007; Winfield *et al.,* 2010; Caudron *et al.,* 2014). Both environmental parameters lead to a decrease of dissolve oxygen level in waters to the point of causing hypoxic events. In fact, high temperatures reduce oxygen solubility and induce stratification of the water column (Jane *et al.,* 2021) while eutrophication promotes algae biomass during warmer months (O’Hare *et al.,* 2018; Wurtsbaugh *et al.,* 2019), resulting in an increase in oxygen concentration during the day and hypoxic events at night (Odum, 1956). Climate change, urbanization and agriculture aggravates these factors (Moss *et al.,* 2011; Le Moal *et al.,* 2019) and aquatic ecosystems are facing an accentuation in the intensity and frequency of these diel hypoxic events (Tyler *et al.,* 2009; Miyamoto *et al.,* 2019). Cyclic hypoxia occurs in lakes (Andersen *et al.,* 2017) and coastal waters (Tyler *et al.,* 2009), but also in rivers, including those in non-urban areas (Blaszczak *et al.,* 2023). Finally, fish may face a higher risk of chronic hypoxia exposure during diel hypoxia since it may be more challenging to evade compared to constant hypoxia (Levin *et al.,* 2009).

Decreased oxygen concentration in the aquatic environment can lead to a reduction of systemic partial pressure of oxygen (PO_2_) and hypoxemia in fish (Farrell and Richards, 2009). The oxygen supply may be preserved through hyperventilation (Perry *et al.,* 2009; Eom and Wood, 2020), increased hematocrit (Jia *et al.,* 2021) or root effect (Rummer and Brauner, 2015). However, if the oxygen supply is insufficient, tissues become hypoxic (Samuel and Franklin, 2008) which leads to a decrease of ATP production through oxidative phosphorylation. To avoid a mismatch between ATP supply and demand, anaerobic glycolysis is activated (Speers-Roesch *et al.,* 2013; Abdel-Tawwab *et al.,* 2019). Fish may also switch into an hypometabolic state and reorganize their metabolism (Nilsson and Renshaw, 2004; Richards, 2009). Multiple metabolic pathways are inevitably affected, notably in the liver (Dahl *et al.,* 2021; Farhat *et al.,* 2021), which is involved in carbohydrate, lipid and protein metabolism (Charlton, 1996; Rui, 2014) and as such, plays a central role in energy metabolism. During hypoxia, carbohydrates and lipids are mobilized to produce ATP while the rate of protein synthesis is depressed to reduce energy demand (Lardon *et al.,* 2013; Cassidy *et al.,* 2018; Li *et al.,* 2018; Cassidy and Lamarre, 2019). Consequently, hypoxia may decrease glycogen and lipid stores (Gracey *et al.,* 2011; Cadiz *et al.,* 2018). By altering fish metabolism, hypoxia may cause an impairment in their reproduction, growth and survival (Pichavant *et al.,* 2001; Landry *et al.,* 2007; Aksakal and Ekinci, 2021).

The return to normoxia following a hypoxic bout could be seen as a welcomed event for fish. However, it is associated with effects similar to ischemia–reperfusion (Li and Jackson, 2002). The return of oxygen causes an oxidative burst (Loor *et al.,* 2011) due to the overproduction of reactive oxygen species (ROS) by mitochondria (Li and Jackson, 2002). Chouchani *et al.* (2016) demonstrated a mechanism that explains this oxidative burst. During the hypoxic phase, the lack of oxygen as the final electron acceptor results in a reduction of the electron transport system (ETS), especially at the level of coenzyme Q. Fumarate then captures the excess electrons and is reduced to succinate, which serves as electron storage, through the succinate dehydrogenase (respiratory complex II) (Chouchani *et al.,* 2016). During reoxygenation, the massive supply of electrons by oxidation of the accumulated succinate causes a reverse transport of electrons to the NADH dehydrogenase (respiratory complex I), resulting in a significant production of ROS and associated damages to lipids, proteins and DNA (Kehrer, 1993).

Numerous studies have investigated the effects of hypoxia on fish, however only a few considered the consequences of both hypoxia and reoxygenation, and most studies focused on hypoxia-tolerant species. The current knowledge about fish responses to cyclic hypoxia is limited, especially considering hypoxia-sensitive species that are the most threatened by such environmental change. As recently outlined, aquatic environment is not static, and we need to take into account the diel variation of its environmental parameters, such as oxygen, temperature and pH, to improve our predictions about the effects of global change on fish (Morash *et al.,* 2018). Previous studies demonstrated that the response to cyclic hypoxia differs from that to constant hypoxia (Borowiec *et al.,* 2015, 2018; Williams *et al.,* 2019; Yao *et al.,* 2022). Furthermore, to our knowledge, the metabolic responses to long-term cyclic hypoxia have only been studied in hypoxia tolerant species (Bera *et al.,* 2017; Borowiec *et al.,* 2018; Zhao *et al.,* 2022). The currently available literature also provide mixed results, depending on the species, on the effects of cyclic hypoxia on growth, and once again, focused mainly on hypoxia-tolerant species (Yang *et al.,* 2013; Dan *et al.,* 2014; Remen *et al.,* 2014; Obirikorang *et al.,* 2020). Currently, only the work of Bourloutski (2022) provides some information on the effects of cyclic hypoxia on Arctic char. Even though Arctic char seems more tolerant to hypoxia when compared to Atlantic salmon, *Salmo salar* (Anttila *et al.,* 2015), previous studies demonstrated its sensitivity to low oxygen levels (McDonald and McMahon, 1977; Gee *et al.,* 1978; Jones *et al.,* 2008). This raises questions about the future of the southernmost wild populations, which will be increasingly exposed to episodes of cyclic hypoxia.

The objective of this study was to characterize the effects of short- and long-term diel cycling hypoxia on growth, physiological condition and energy metabolism of Arctic char (*Salvelinus alpinus*) during warmer months of the year. Specifically, we exposed warm acclimated fish to 30 days of cyclic hypoxia and measured growth parameters, hematocrit, rate of protein synthesis and triglycerides reserves (TAG) in the liver, carbohydrate reserves in white muscle and characterized the liver metabolome. We hypothesized that, when facing multiple days of cyclic hypoxia, Arctic char alters its energy metabolism and energy allocation to ensure ATP supply meets ATP demand and control oxidative stress. We predicted that Arctic char experiencing chronic cyclic hypoxia would have diminished energy stores and impaired energy allocation reflected by curtailed growth rate and lower condition indices compared to control (normoxic) fish. We also predicted a compensation of the rate of protein synthesis during the reoxygenation periods to catch-up for the synthesis delay accrued during hypoxic periods. Finally, we predicted that these metabolic adjustments would be reflected in the fish metabolome with changes in amino acids, lipids and carbohydrates pathways.

## Materials and Methods

### Animals

Arctic char (Fraser strain) were obtained from the aquaculture facility of the research center Valorēs Inc. (Shippagan, NB, Canada). The fish were kept in 250 L tanks with air saturated dechlorinated water supplied at approximately 16°C, in a 14:10 light cycle (220 lux). In the wild, this species sometimes experience temperatures of 20°C (Gilbert *et al.,* 2016). The fish were fed *ad libitum* three times a week with a commercial diet (Nutra RC 80A, Skretting, Bayside, NB, Canada). All animal experiments were approved by the Institutional Animal Care Committee (UdeM 19–12). At least 2 weeks before the experiment, the fish were anesthetized in a 50-mg.L^−1^ benzocaine solution and individually tagged with 8 mm × 1.4 mm FDX PIT tags (Oregon RFID, Portland, USA) injected in the abdominal cavity.

### Experimental design

For the cyclic hypoxia experiments, we used a recirculation system composed of two independent units (multi-stressor system, Aquabiotech Inc., QC, Canada). Experiments were conducted at 16°C to be consistent with the simulation of a hypoxic event that is more likely to occur during the warm season. A total of 240 fish (16.56 ± 0.21 g, 12.90 cm ±0.06) were randomly transferred into ten 9-L tanks (24 fish per tank). The fish were fed 0.5% biomass each day and fasted 24 hours prior sampling. No difference was observed in food intake between tanks. After 14 days of acclimation in air-saturated water, half of the tanks were exposed to cyclic hypoxia for 30 days. The oxygen concentration was reduced to 20% air saturation (1,97 mg.L^−1^ of oxygen) within about 2 hours between 8:00 pm and 6:00 am (hypoxic phase) by injecting N_2_. This oxygen concentration was selected based on a previous experiment, carried out on the same strain of Arctic char, which revealed that fish tended to lose equilibrium when kept at 15% air saturation at 16°C during 2 hours (Bourloutski, 2022). The oxygen concentration was then maintained at 100% air saturation (9,87 mg.L^−1^ of oxygen) for the rest of the day (normoxic phase). In each tank, four fish were sampled at 9:00 am on Days 1, 5, 10 and 30 (D1, D5, D10, D30). The tank was considered as the experimental unit (n = 5 per condition). The density of fish in each tank was kept constant using a sliding plexiglass wall to limit the formation of dominance hierarchy. The fish were weighed and measured after being euthanized by a blow to the head. A blood sample was collected with a needle and syringe from the caudal vein and centrifuged at 1000 × *g* for 5 minutes at 4°C in a hematocrit capillary tube. Liver, heart, digestive tract and white muscle were quickly dissected, weighed (excepted white muscle), frozen in liquid nitrogen and stored at −80°C until further experiments. The hepatosomatic index, visceral index and relative heart mass were determined according to the following equations:


(1) \begin{equation*} \mathrm{Hepatosomatic}\ \mathrm{index}\ \left(\%\right)=\frac{\mathrm{liver}\ \mathrm{mass}}{\mathrm{body}\ \mathrm{mass}}\times 100 \end{equation*}

(2) \begin{equation*} \mathrm{Visceral}\ \mathrm{index}\ \left(\%\right)=\frac{\mathrm{viscera}\ \mathrm{mass}}{\mathrm{body}\ \mathrm{mass}}\times 100\end{equation*}

(3) \begin{equation*} Relative\ heart\ mass\ \left(\%\right)=\frac{heart\ mass}{body\ mass}\times 100\end{equation*}

On Days 0, 10, 20 and 30 (D0, D10, D20, D30), all fish were anesthetized in a 50 mg.L^−1^ benzocaine solution, weighed and measured. The specific growth rate (SGR) in length and mass, as well as the condition factor (CF), were calculated according to the following equations:


(4) $$ \small\begin{equation*} \mathrm{SGR}\ \mathrm{in}\ \mathrm{lenght}\ \left(\%.{\mathrm{day}}^{-1}\right)=\frac{\ln \left({\mathrm{lenght}}_{\mathrm{t}2}\right)-\ln \left({\mathrm{lenght}}_{\mathrm{t}1}\right)}{\Delta \mathrm{days}}\times 100\end{equation*}

(5) $$ \small\begin{equation*} \mathrm{SGR}\ \mathrm{in}\ \mathrm{mass}\ \left(\%.{\mathrm{day}}^{-1}\right)=\frac{\ln \left({\mathrm{mass}}_{\mathrm{t}2}\right)-\ln \left({\mathrm{mass}}_{\mathrm{t}1}\right)}{\Delta \mathrm{days}}\times 100\end{equation*}

(6) \begin{equation*} \mathrm{Condition}\ \mathrm{factor}=\frac{\mathrm{mass}}{{\mathrm{lenght}}^3}\times 100 \end{equation*}

(7) \begin{align*} \mathrm{Condition}\ &\mathrm{factor}\ \mathrm{variation}\ \left(\%.{\mathrm{day}}^{-1}\right)\nonumber\\&=\frac{\frac{{\mathrm{condition}\ \mathrm{factor}}_{\mathrm{t}2}-{\mathrm{condition}\ \mathrm{factor}}_{\mathrm{t}1}}{{\mathrm{condition}\ \mathrm{factor}}_{\mathrm{t}1}}}{\Delta \mathrm{days}}\times 100\end{align*}

Only fish that were kept throughout the experiment were included in the calculation of morphological parameters.

### Energy stores

#### White muscle glucose, glycogen and lactate

Approximately 40 mg of frozen white muscle (two fish processed per tank per condition) were homogenized by ultrasonication (Q55 Sonicator, Qsonica Inc., USA) in five volumes of 1 M perchloric acid (PCA; A228 Fisher Scientific, USA). The samples were neutralized by adding KHCO_3_. Glucose was assayed in a 250-mM imidazole buffer supplemented with 5 mM MgSO_4_·7H_2_O, 10 mM ATP, 1 mM NADP^+^, pH 7.8. Following the addition of G6PDH and hexokinase in excess, absorbance at 340 nm was recorded. Glucose concentration was determined from a standard curve and reported in μmol.g^−1^ tissue. For glycogen, the neutralized samples were incubated 2 hours at 37°C with amyloglucosidase (50 U.mL^−1^) in acetate buffer (0.4 M, pH 4.8). Glucose concentration was then determined as above, and glycogen concentration was reported in μmol glycosyl units per g tissue. Lactate concentration was determined as described in Brandt *et al.* (1980). Briefly, samples or standards were added to 10 volumes of assay buffer (320 mM glycine, 320 mM hydrazine, 2.4 mM NAD^+^, pH 9.5). Lactate concentration was determined by reading absorbance at 340 nm after a 150-minute incubation at 25°C with LDH in excess. Results are presented as means ± sem and expressed as μmol.g^−1^ tissue.

#### Liver triglycerides

Lipids were extracted from approximately 20 mg of frozen liver (two fish processed per tank per condition) using chloroform/methanol extraction (Bligh and Dyer, 1959). Extracted lipids were resuspended in a 95% ethanol. Lipid concentration was determined using a commercial kit (Infinity Reagent, Thermo Scientific, USA) and reported as means ± sem and expressed as μmol glycerol units per g tissue. For all energy store assays, the intra- and inter-assay variation was always 5% or lower.

### Protein synthesis

The fractional rate of protein synthesis (*Ks*) was determined following the flooding dose method developed by Garlick *et al.* (1980) and modified according to Lamarre *et al.* (2015). Three fish per tank per condition received an intraperitoneal injection of a 150 mM phenylalanine (PHE) solution containing 50% ring-[D_5_]-L-phenylalanine ([D_5_]-PHE, 98%, Cambridge Isotope Laboratories, Inc. Andover, USA) at a dose of 1 mL per 100 g of body mass and returned to their tanks for an incorporation period of three hours. The fish were then sacrificed, and the liver was flash frozen and kept at −80°C. Approximately 20 mg of frozen tissue were homogenized in 0.2 M PCA by ultrasonication and centrifuged. The supernatant, which contains protein-free amino acid pool (FP), was collected and stored at −20°C. The pellet containing the protein pool (PP) was washed three times in 0.2 M PCA. The pellet was finally washed with 1 mL of acetone to remove excess lipids before being hydrolyzed in 6 M HCl at 110°C for 18 hours. PP and FP phenylalanine was extracted using solid phase extraction as described in Lamarre *et al.* and Cassidy *et al.* (Lamarre *et al.,* 2015; Cassidy *et al.,* 2016). The extracted amino acids were derivatized using pentafluorobenzyl bromide (PFBBr). Briefly, 50 μL of sample were incubated 45 minutes at 60°C with 20 μL of phosphate buffer (0.5 M, pH 8.0) and 130 μL of PFBBr (100 mM in acetone). Derivatized amino acids were extracted in 330 μL of hexane, transferred to low volume inserts before analysis. GC–MS analyses were performed using an Agilent gas chromatograph (model 6890N) interfaced to a single quadrupole inert mass selective detector (MSD, model 5973) as previously described by Lamarre *et al.* (2015). *Ks* (%.day^−1^) was calculated according to the Eq. 8:


(8) \begin{equation*} Ks\ \left(\%.{\mathrm{day}}^{-1}\right)=\frac{S_b}{S_a}\times \frac{1440}{t}\times 100 \end{equation*}

where *S*_a_ is the FP enrichment (*S*_a_ = [D_5_] − PHE/(PHE + [D_5_] − PHE)), *S*_b_ the PP enrichment (*S_b_* = [D_5_] − PHE/(PHE + [D_5_] − PHE)) and *t* the incorporation time in min.

### Metabolite extraction and quantification

Approximately 50 mg of frozen liver from non-injected fish (one fish processed per tank per condition) were homogenized in 250 μL of cold acetonitrile with an ultrasound homogenizer on ice. A 250-μL cold water was then added and the solution was homogenized by sonication on ice. After a 5-minute 10 000 × *g* centrifugation at 4°C, 400 μL of the supernatant containing hydrophilic metabolites were transferred to a borosilicate tube and evaporated 1 hour under a nitrogen stream. The samples were stored at −80°C until further analysis.

Just before ^1^H NMR analysis, samples were resuspended in 900 μL of D_2_O and 100 μL of 4,4-dimethyl-4-silapentane-1-sulfonic acid (DSS, internal calibrator, 0.5 mM final concentration). Approximately 700 μL were transferred to a 5-mm NMR tube. The ^1^H-NMR spectra were recorded on a Bruker Advance III 400 MHz spectrometer at 298K. Analyses were performed with the *noesypr1d* pulse sequence. One-dimensional spectra were obtained after 128 scans of 64 000 data points. The spectral width was set at 12 KHz, the acquisition time at 6.6 seconds and the recycle delay at 1 second per scan. Correction and calibration of the spectra were performed using the Chenomx NMR Processor (Chenomx Inc., Edmonton, AB, Canada) and analyzed using the Chenomx NMR Profiler (Chenomx Inc., ) and the Human Metabolome Database (HMDB) (Wishart *et al.,* 2018).

### Statistics

Statistical analyses were performed with the R software (R Core Team, 2023). Data were fitted to a mixed linear model with treatments (normoxia, cyclic hypoxia) and sampling day (or periods) as fixed factors and experimental tank as random factor. Normality and homoscedasticity of residuals were checked with a Shapiro–Wilk and Agostino tests and a Levene test, respectively. When necessary, Box-Cox or rank transformation was applied. Two-way ANOVAs were performed to test the effects of treatment and sampling day (or periods) on growth, CF variation, energy store or *Ks*. When a significant interaction was detected, a *post hoc* test was performed using the least squares mean method with p-values adjusted by the Tukey method. The significance threshold was set to *p* < 0.05. A two-sample t-test was performed to test the effect of treatment on SGR and CF variation after 30 days of exposure.

Statistical analyses of the metabolomics data were performed using MetaboAnalyst 5.0 (https://www.metaboanalyst.ca/). Metabolite concentrations were analyzed using a partial least squares discriminant analysis (PLS-DA) (Chong *et al.,* 2019) to determine the variable importance to the projection (VIP) score. Data were log transformed and - normalized (i.e. mean-centered and divided by the standard deviation of each variable) was applied in order to minimize possible differences in concentration between samples. Metabolites that were assigned a VIP score > 1.0 were considered important in the PLS-DA model (Chong *et al.,* 2019) and were further analyzed for specific differences (two-sample t-test).

## Results

### Growth

**Figure 1:**
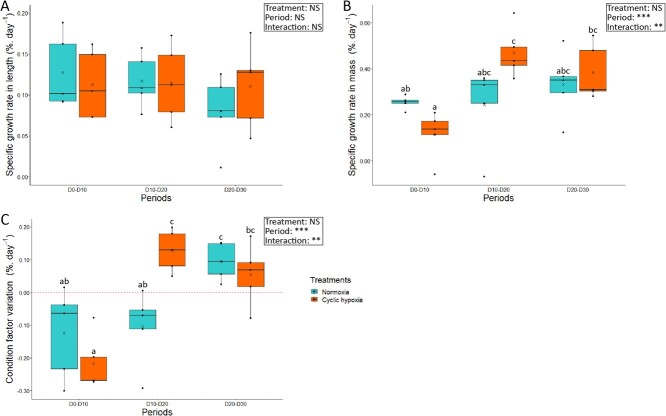
Specific growth rate in length (A) and mass (B) and CF variation (C) of Arctic char for each ten-day period, between day 0 and day 30, after fish were either maintained in normoxia or exposed to cyclic hypoxia (n = 5). Box plots indicate the median (—), the mean (×), 25^th^ and 75^th^ percentiles (box), 95% range (|) and each observation (●). Two-way ANOVA results are shown as NS (non-significant), ^**^ (*p* < 0.01), ^***^ (*p* < 0.001). Different letters indicate significant differences between groups (*post-hoc* test, p-value < 0.05).

We monitored several growth parameters of fish exposed to 30 days of normoxia or cyclic hypoxia. Average SGR in length and mass were not significantly different between the two groups at the end of the experiment (Supplementary Table 1). The CF variation remained overall stable and did not differ between treatments (Supplementary Table 1). Analyzing the results using periods of 10 days revealed that SGR in length was stable (Fig. 1A), while SGR in mass changed significantly over time (*F*_*2,16*_ = 20.39, *p* < 0.001; Fig. 1B). There was no significant difference in the normoxic group (Supplementary Table 2), however the cyclic hypoxia group gained less mass during the first 10 days (D0–D10) than during the D10-D20 (*p* < 0.001) and D20–D30 periods (*p* < 0.01).

**Table 1:** Energy stores and lactate concentration of Arctic char exposed to 1, 5, 10 and 30 days of cyclic hypoxia or normoxia

	**Normoxia**				**Cyclic hypoxia**				**Two-way Anova (F-values)**		
	D1	D5	D10	D30	D1	D5	D10	D30	Treatment Num *df* = 1 Den *df* = 8	Sampling day Num *df* = 3 Den *df* = 24	Interaction Num *df* = 3 Den *df* = 24
**White muscle**											
Glucose ^T^ (μmol.g^−1^)	3.79 *± 0.50*	3.50 *± 0.18*	3.65 *± 0.30*	4.23 *± 0.65*	3.48 *± 0.24*	3.52 *± 0.33*	3.92 *± 0.39*	4.25 *± 0.52*	0.00	0.92	0.11
Glycogen (μmol.g^−1^)	14.58 *± 1.92*	14.14 *± 0.73*	11.56 *± 1.28*	15.40 *± 1.76*	11.89 *± 0.89*	11.37 *± 1.23*	15.29 *± 1.63*	18.00 *± 2.07*	0.03	3.31^*^	3.00
Lactate (μmol.g^−1^)	34.61 *± 5.51*	32.88 *± 1.12*	35.29 *± 2.32*	28.67 *± 3.47*	36.59 *± 4.80*	37.20 *± 3.52*	32.64 *± 3.39*	32.31 *± 2.77*	0.42	0.88	0.41
**Liver**											
Triglycerides (μmol.g^−1^)	4.24 *± 0.42*	4.67 *± 0.27*	4.27 ± 0.74	4.20 *± 0.76*	3.87 *± 0.30*	4.25 *± 0.39*	5.51 *± 0.58*	2.94 *± 0.41*	0.33	2.40	2.08
Hepatosomatic index (%)	1.04 ± 0.08 ^a,b^	1.01 ± 0.09 ^a,b^	1.16 ± 0.09 ^a,b^	1.01 ± 0.05 ^a,b^	0.97 ± 0.05 ^a^	1.22 ± 0.08 ^b^	1.11 ± 0.04 ^a,b^	1.04 ± 0.03 ^a,b^	0.12	5.24^**^	4.89^**^
**Viscera**											
Visceral index (%)	5.17 *± 0.31*	5.50 *± 0.42*	4.97 *± 0.18*	4.84 *± 0.23*	5.50 *± 0.37*	5.10 *± 0.37*	5.23 *± 0.25*	4.93 *± 0.25*	0.03	4.21^*^	2.61

Data is means ±*sem*, n = 5; ^T^ data Box Cox transformation applied; ^*^p < 0.05, ^**^ (*p* < 0.01). Different letters indicate significant differences (post-hoc test, *p* < 0.05).

The CF variation was significantly affected by the period (*F*_*2,16*_ = 19.09, *p* < 0.001; Fig. 1C) similarly to SGR in mass. Both normoxia and cyclic hypoxia fish had a negative CF variation between D0 and D10. CF then increased between D10 and D20 for fish exposed to cyclic hypoxia compared to the first 10 days (*p* < 0.001). However, for fish in normoxia, CF only improved between D20 and D30 (*p* < 0.05) comparatively to the first 10 days. Furthermore, CF variation was significantly higher during cyclic hypoxia between D10 and D20 (*p* < 0.05) compared to the normoxic group.

### Energy stores

The energy stores were not affected by cyclic hypoxia. In white muscle, neither glucose nor glycogen concentrations differed between the control and fish exposed to cyclic hypoxia (Table 1). Moreover, lactate was also stable.

No differences were also detected in liver triglyceride concentrations (Table 1). The hepatosomatic index was unchanged between groups for the duration of the experiment, except for a slight increase between D1 and D5 in the cyclic hypoxia group. The visceral index was also not affected by the treatment throughout the experiment (Table 1).

### Oxygen supply

Overall, exposure to cyclic hypoxia resulted in a slightly higher hematocrit compared to normoxia (*F*_1,8_ = 7.76, *p* < 0.05) but the number of hypoxia cycles experienced by the fish did not affect the hematocrit (Supplementary Fig. 1). Moreover, relative heart mass remained unaffected by cyclic hypoxia (*F*_1,8_ = 0.01, *p* = 0.91).

### Protein synthesis

**Figure 2:**
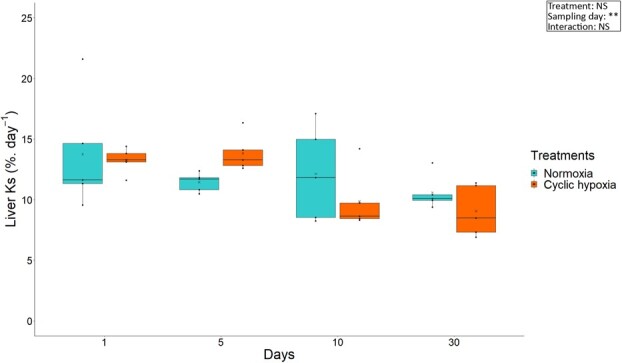
Liver *Ks* of Arctic char at days 1, 5, 10 and 30 after fish were either maintained in normoxia or exposed to cyclic hypoxia (n = 5). Box plots indicate the median (—), the mean (×), 25^th^ and 75^th^ percentiles (box), 95% range (|) and each observation (●). Two-way ANOVA results are shown as NS (non-significative), ^**^ (*p* < 0.01).

The rate of protein synthesis of the liver was not affected by cyclic hypoxia (Fig. 2). No compensation of *Ks* during reoxygenation occurred in the liver at the beginning of the exposure to cyclic hypoxia. Liver *Ks* decreased slightly over time in both groups (*F*_3,24_ = 5.67, *p* < 0.01).

### Metabolomic analysis

A total of 68 metabolites were identified. Metabolites for which identification or quantification was not reliable, and metabolites from potential exogenous sources were removed. Finally, 49 metabolites were included in the analysis (Supplementary Table 3). Metabolite concentrations were first analyzed over time in each treatment group (*i.e.* normoxia and cyclic hypoxia) using a PLS-DA. In normoxia, the two main components explained 10.6% and 7.6% of the total variation, respectively (Supplementary Fig. 2A). This analysis revealed that D1 and D5 were more similar, while D10 differed slightly from the previous days. However, D30 was clearly separated from the early days. Twenty metabolites contributed to this metabolic change (Supplementary Fig. 2B, VIP score > 1.0 for component 1). However, this model had low predictability (Q2) and accuracy (Supplementary Table 4). During cyclic hypoxia, components 1 and 2 explained 17.7% and 9.4% of the variance, respectively (Supplementary Fig. 2C). PLS-DA separated each sampling day with better segregation compared to the normoxic group. Twenty metabolites mainly drove this signature (Supplementary Fig. 2D, VIP score > 1 for component 1) with moderate accuracy and predictability (Supplementary Table 4). Considering that these changes over time largely resulted from the experimental conditions, we decided to focus on the differences between treatments at each sampling day.

**Figure 3:**
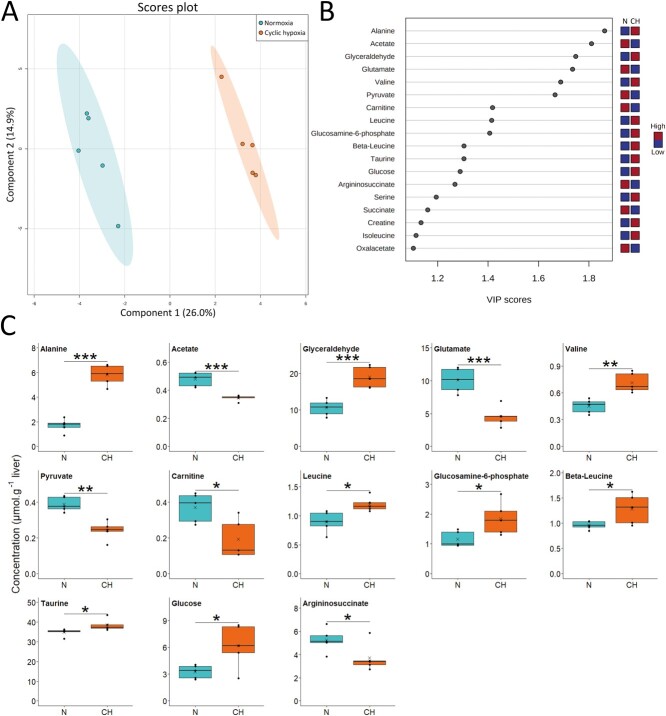
Metabolite profile in liver of Arctic char at day 1 after fish were either maintained in normoxia or exposed to cyclic hypoxia (n = 5): A) PLS-DA 2D score plots of liver metabolites. Components 1 and 2 represent variance proportion and ellipses correspond to 95% confidence intervals for each treatment group. B) Variable importance in projection (VIP) scores of PLS-DA component 1 for liver metabolites which drive metabolic profile differentiation between the normoxic (N) and cyclic hypoxic (CH) groups (VIP score > 1). Relative concentrations of corresponding metabolites are indicated by colored boxes on the right. C) Concentration of metabolites which differ significatively between normoxic (N) and cyclic hypoxic (CH) fish. Box plots indicate the median (—), the mean (×), 25^th^ and 75^th^ percentiles (box), 95% range (|) and each observation (●). Two sample t-test results are shown as ^*^ (*p* < 0.05), ^**^ (*p* < 0.01), ^***^ (*p* < 0.001).

Following one hypoxia-reoxygenation cycle, we observed a large separation between the normoxia and cyclic hypoxia groups on the first component, which represented 26.0% of the total variation (Fig. 3A). This signature was driven by 18 metabolites (Fig. 3B). This model had high accuracy and predictability (Supplementary Table 4). The main change was a 3.4-fold increase in alanine concentration in cyclic hypoxia (Fig. 3C). Furthermore, liver of fish exposed to cyclic hypoxia accumulated branched-chain amino acids (BCAAs; leucine, isoleucine and valine) as well as beta-leucine, taurine, glucose, glyceraldehyde and glucosamine-6-phosphate. Glutamate, carnitine, argininosuccinate and pyruvate levels were all significantly lower in the cyclic hypoxia group. Moreover, the concentrations of succinate and oxaloacetate were slightly lower but did not reach significance in the same group.

**Figure 4:**
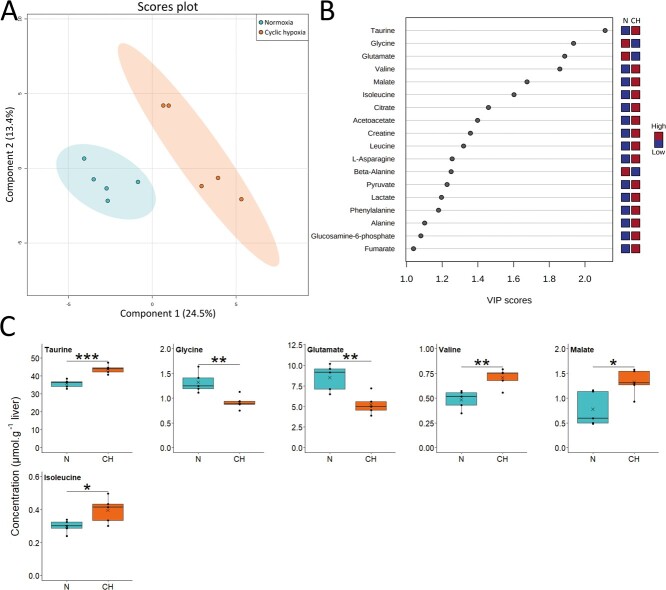
Metabolite profile in liver of Arctic char at day 5 after fish were either maintained in normoxia or exposed to cyclic hypoxia (n = 5): A) PLS-DA 2D score plots of liver metabolites. Components 1 and 2 represent variance proportion and ellipses correspond to 95% confidence intervals for each treatment group. B) Variable importance in projection (VIP) scores of PLS-DA component 1 for liver metabolites which drive metabolic profile differentiation between the normoxic (N) and cyclic hypoxic (CH) groups (VIP score > 1). Relative concentrations of corresponding metabolites are indicated by colored boxes on the right. C) Concentration of metabolites which differ significatively between normoxic (N) and cyclic hypoxic (CH) fish. Box plots indicate the median (—), the mean (×), 25^th^ and 75^th^ percentiles (box), 95% range (|) and each observation (●). Two sample t-test results are shown as ^*^ (*p* < 0.05), ^**^ (*p* < 0.01), ^***^ (*p* < 0.001).

Five days of cyclic hypoxia resulted in a similar pattern. Differentiation of metabolic profile between treatments was also obvious with the first component explaining 24.5% of the variation (Fig. 4A). Eighteen metabolites led this pattern (Fig. 4B). The fish exposed to cyclic hypoxia had lower concentrations of glutamate and higher BCAAs than the normoxic fish (Fig. 4C). Cyclic hypoxia also led to a significant decrease in glycine concentration while the accumulation of glucose observed at D1 disappeared. Finally, the concentrations of malate, citrate and fumarate were all elevated in the cyclic hypoxia fish.

**Figure 5:**
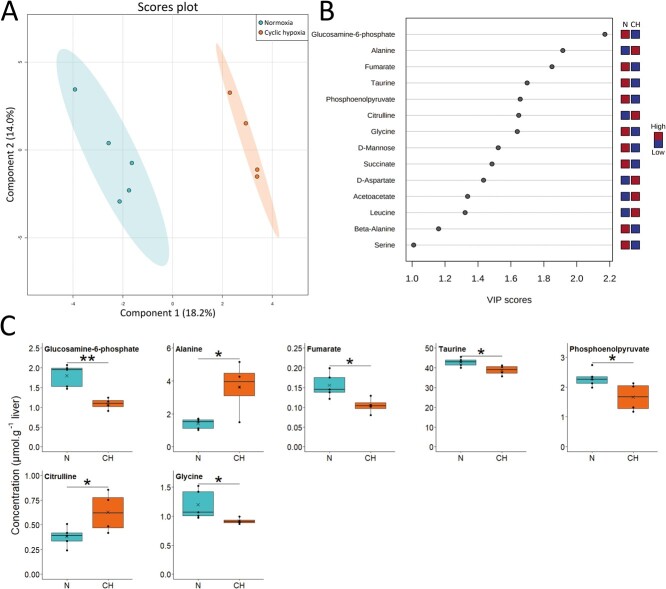
Metabolite profile in liver of Arctic char at day 10 after fish were either maintained in normoxia (n = 5) or exposed to cyclic hypoxia (n = 4): A) PLS-DA 2D score plots of liver metabolites. Components 1 and 2 represent variance proportion and ellipses correspond to 95% confidence intervals for each treatment group. B) Variable importance in projection (VIP) scores of PLS-DA component 1 for liver metabolites which drive metabolic profile differentiation between the normoxic (N) and cyclic hypoxic (CH) groups (VIP score > 1). Relative concentrations of corresponding metabolites are indicated by colored boxes on the right. C) Concentration of metabolites which differ significatively between normoxic (N) and cyclic hypoxic (CH) fish. Box plots indicate the median (—), the mean (×), 25^th^ and 75^th^ percentiles (box), 95% range (|) and each observation (●). Two sample t-test results are shown as ^*^ (*p* < 0.05), ^**^ (*p* < 0.01).

After 10 days of exposure to cyclic hypoxia, the differences between normoxic and cyclic hypoxic fish were not as strong as the first days, as the variation explained by the first component (18.2%) was lower compared to D1 and D5 (26% and 24.5%, respectively) (Fig. 5A). Separation between groups was mainly due to 14 different metabolites (Fig. 5B). Alanine and glycine concentrations remained high and low, respectively, after 10 days of cyclic hypoxia (Fig. 5C). The effect of cyclic hypoxia on glutamate and BCAAs was no longer detected despite a slightly elevated leucine concentration. Fumarate and succinate concentrations were lower in the cyclic hypoxia group.

**Figure 6:**
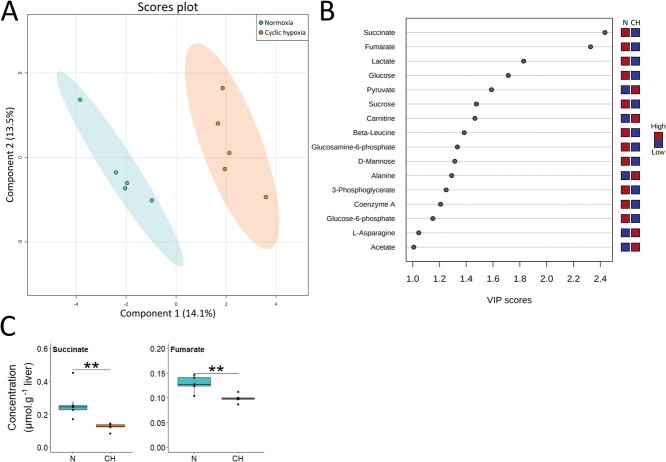
Metabolite profile in liver of Arctic char at day 30 after fish were either maintained in normoxia or exposed to cyclic hypoxia (n = 5): A) PLS-DA 2D score plots of liver metabolites. Components 1 and 2 represent variance proportion and ellipses correspond to 95% confidence intervals for each treatment group. B) Variable importance in projection (VIP) scores of PLS-DA component 1 for liver metabolites which drive metabolic profile differentiation between the normoxic (N) and cyclic hypoxic (CH) groups (VIP score > 1). Relative concentrations of corresponding metabolites are indicated by colored boxes on the right. C) Concentration of metabolites which differ significatively between normoxic (N) and cyclic hypoxic (CH) fish. Box plots indicate the median (—), the mean (×), 25^th^ and 75^th^ percentiles (box), 95% range (|) and each observation (●). Two sample t-test results are shown as ^**^ (*p* < 0.01).

Finally, after a full month of cyclic hypoxia (D30), the differences between the two groups was down to only 14.1% on the first component (Fig. 6A). Among the 16 metabolites with VIP scores greater than 1, two metabolites, succinate and fumarate, especially drove the pattern (Fig. 6B). Both had significantly lower concentrations in fish exposed to cyclic hypoxia (Fig. 6C). Despite the absence of significant differences, the succinate:fumarate ratio had a tendency to decrease after 30 days of cyclic hypoxia (Supplementary Fig. 3A). Another interesting ratio tendency was pyruvate:lactate (Supplementary Fig. 3B) that showed an upward trend with time in cyclic hypoxia. Conversely, a downward trend was observed in normoxia.

## Discussion

This study investigated the effects of cyclic hypoxia on Arctic char growth, energy stores and liver metabolome. We hypothesized that cyclic hypoxia would induce a modulation of fish metabolism and a change in energy management that would eventually impair energy allocation toward growth. In support of our prediction, we found a metabolic alteration in the liver of Arctic char. However growth and energy stores were preserved. Arctic char were able to cope with a month of cyclic hypoxia. The fish were exposed to conditions that were harsher than what is expected to find in the wild and yet, they were able to maintain their physiological condition through efficient adjustments of their phenotype.

### Resilience of growth and energy stores after 1 month of cyclic hypoxia

Arctic char were more resistant to cyclic hypoxia than anticipated. After 1 month of cycling, the growth parameters and physiological conditions remained very similar to those of the control group. During the first 10 days of cyclic hypoxia, the SGR in mass of fish tend to be lower than control with a very limited impact on CF variation. An impairment in growth performance due to cyclic hypoxia was previously observed in salmon (Remen *et al.,* 2014), catfish (Yang *et al.,* 2013) and flounder (Davidson *et al.,* 2016). In salmon, growth impairment is explained by a decreased food intake but not by food utilization, unlike in catfish (Yang *et al.,* 2013; Remen *et al.,* 2014). A similar phenomenon was observed in seabass and turbot exposed to chronic hypoxia (Pichavant *et al.,* 2001). In the present experiment, feed ration was adjusted to 0.5% biomass^−1^∙day^−1^ in both groups and no uneaten food was observed. The maintenance of food intake may partly explain the lack of significant impact of cyclic hypoxia on Arctic char growth in this study.

After 10 days of cyclig hypoxia, SGR in mass tends to be higher than in the control group, which may be indicative of compensatory growth. This phenomenon has already been observed in salmon exposed to cyclic hypoxia but only after a 1-month recovery period in normoxia (Remen *et al.,* 2014). Foss *et al.* (2002) reported a recovery from SGR in spotted wolffish exposed to moderate hypoxia for 3 months. These authors also observed compensatory growth for this species during the recovery period in normoxia (Foss and Imsland, 2002). However, while compensatory growth may be considered beneficial (Ali *et al.,* 2003; Mangel and Munch, 2005), it often comes with its own costs (Metcalfe and Monaghan, 2001). But if cyclic hypoxia elicited compensatory growth, it was only for a short period, with a low intensity. Overall, cyclic hypoxia had little impact on the growth rate of Arctic char, indicating that this species appears resilient to this environmental stressor. It is also possible that energy allocation to growth is preserved at the expense of other non-vital processes.

Contrary to our prediction but consistent with the growth results, tissue energy reserves remained stable during the 30 days of cyclig hypoxia. It is interesting to note that Borowiec *et al.* (2018) also observed that chronic cyclic hypoxia induced no change in free glucose, glycogen and lactate concentrations in skeletal muscle of killifish, a hypoxia-tolerant species. Concerning hepatic TAG levels, Li *et al.* (2018) found that TAG levels decrease in the liver of Nile tilapia, but only after 4 weeks of chronic constant hypoxia, and that acute hypoxic stress was insufficient to induce the use of TAG in the liver. According to our data, TAG and lipid reserves are not used as fuel in Arctic char experiencing cyclic hypoxia.

### Limited increase of oxygen supply capacity

As expected, we observed a slightly higher hematocrit under cyclic hypoxia, which may have been coupled with a possible increase in ventilation (although not specifically measured here), thus improving oxygen uptake and delivery to tissues. That would have helped to maintain aerobic production of ATP and so reduce the impact on growth and energetic reserves despite hypoxia events. However, the lack of change in relative heart mass suggested that there was no improvement in cardiac capacity in response of cyclic hypoxia.

### No evidence of protein synthesis debt

The rate of hepatic protein synthesis has been shown to curtail during acute hypoxia in Arctic char (Cassidy and Lamarre, 2019). The liver plays a central role in energetic processes, particularly through its involvement in protein metabolism (Charlton, 1996). Therefore, we expected that a compensation of the protein synthesis activity would occur immediately after reoxygenation. We did not observe such a response, suggesting that Arctic char do not accumulate a “protein synthesis debt” during hypoxia. Although we did not detect significant changes in the rate of protein synthesis, liver metabolism was profoundly affected by cyclic hypoxia, as revealed by metabolomic analysis.

### Metabolite changes in response to cyclic hypoxia

#### Acute stress response

**Figure 7:**
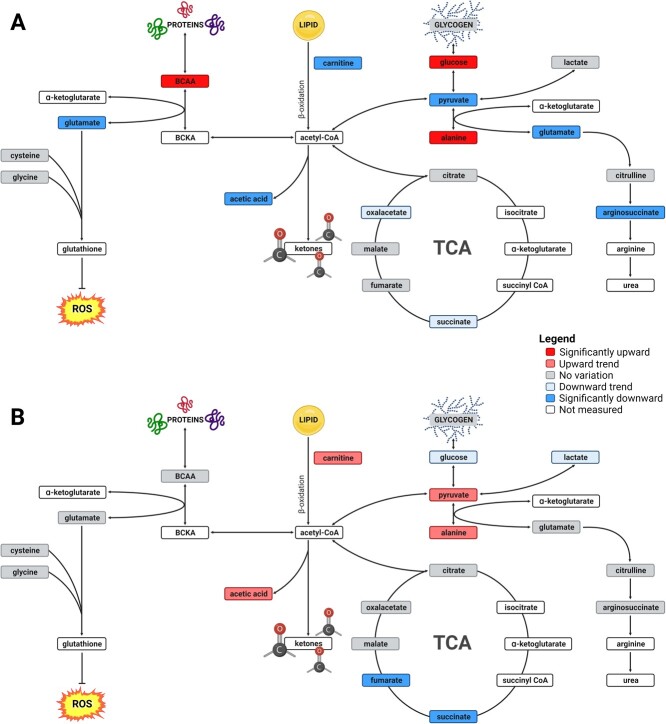
Effects of cyclic hypoxia on the relative change of hepatic metabolic pathway intermediate concentrations in Arctic char at day 1 (A) and day 30 (B) compared to the control group (normoxia). At day 1, several significantly changes were observed in amino acid, carbohydrate and lipid metabolism metabolites. At day 30, only succinate and fumarate concentrations were significantly lower compared to the control group. Created with BioRender.com.

As predicted, the first hypoxia–reoxygenation cycle caused a profound metabolic reorganization in the Arctic char liver (Fig. 7A). Amino acid, carbohydrate and lipid metabolisms were particularly impacted. The amino acids accumulated in large proportions in the liver of fish exposed to hypoxia. Branched chain amino acids accumulation (leucine, isoleucine and valine) suggested an increase in protein catabolism combined with a decrease in protein synthesis, as previously described in Arctic char liver during hypoxia (Cassidy and Lamarre, 2019). These amino acids have also been shown to accumulate during anoxia and after up to three hours of reoxygenation in the liver of crucian carp, before decreasing after a full day of recovery (Dahl *et al.,* 2021).

Alanine is the amino acid that shows the most notable changes. It likely accumulated during hypoxia or anoxia in Siberian wood frog, pond slider and carp (Lardon *et al.,* 2013; Bundgaard *et al.,* 2019; Shekhovtsov *et al.,* 2020; Dahl *et al.,* 2021). In crucian carp tissues, alanine levels remained high up to three hours after reoxygenation (Dahl *et al.,* 2021). Our results suggest the activation of the glucose-alanine cycle, also known as the Cahill cycle (Felig, 1973). Alanine, as a product of glycolysis and protein catabolism in muscle, is exported to the liver to be transaminated into pyruvate. This pyruvate is then reduced into glucose, which is exported to muscle to sustain glycolysis. Accumulated alanine may also be oxidized and may greatly contribute to liver energy metabolism. Jubouri *et al.* (2021) suggested that when available in high concentrations, alanine is the preferred oxidative fuel in trout instead of glucose. An alternative hypothesis supported by Bundgaard *et al.* (2019), is that the lack of oxygen induces a reversal of the TCA cycle. Pyruvate would be therefore aminated into alanine to generate α-ketogluratate from glutamate. However, our metabolite data did not fully supported this phenomenom in Artic char, but this is probably due to the fact that sampling was carried out during reoxygenation. The most parsimonious explanation for our results is thus that alanine is accumulated in the liver during hypoxia due to muscle exportation and α-ketoglutarate synthesis. During reoxygenation, this alanine is transaminated into pyruvate, which in turn forms glucose via gluconeogenesis, providing an explanation for the concomitant higher hepatic glucose concentration.

As mentioned above, glutamate concentration is much lower during acute cyclic hypoxia. Usually, glutamate concentration is maintained or increased in tissues during hypoxia and anoxia (Shekhovtsov *et al.,* 2020; Dahl *et al.,* 2021) except in the brain, where it generates the neurotransmitter GABA (Dahl *et al.,* 2021). Dahl *et al.* (2021) observed a decrease in glutamate concentration in carp liver after a recovery period of one day, but this point was not further discussed. Glutamate is a key metabolite in many metabolic processes. As mentioned, it can supply α-ketoglutarate to the TCA cycle but can also be used to synthesize glutathione, which provides protection against oxidative damages during reoxygenation (Fang *et al.,* 2002). However, we did not detect changes in l-cysteine concentration, another amino acid involved in glutathione synthesis. Glutamate is also an important ammonia carrier and can contribute to its excretion in liver through ureagenesis (Li *et al.,* 2020). Although it is not the main pathway for ammonia excretion in fish (Wilkie, 2002), glutamate depletion coupled with the decrease in argininosuccinate seem to indicate an eventual elimination of ammonia through urea production. An increase in uric acid and urea in anoxic carp liver previously reported by Dahl *et al.* (2021) may support this hypothesis.

Concerning lipid metabolism, the decline in carnitine levels observed in cyclic hypoxia suggests a decreased β-oxidation capacity, which is also substantiated by decreased acetate levels, a by-product of this pathway (Yamashita *et al.,* 2001). Liu *et al.* (2014) demonstrated that hypoxia induced an inhibition of the β-oxidation and promoted lipid accumulation in mouse liver and human hepatocytes (Liu *et al.,* 2014). According to Sun *et al.* (2020)*,* who observed this pattern in largemouth bass, this strategy may help prepare the organism for an eventual long-term hypoxic event (Sun *et al.,* 2020).

In general, these observations suggested that the energetic metabolism was under pressure during the first hypoxia-reoxygenation cycle. In addition to the decreased glutamate levels and the accumulation of alanine observed in fish exposed one day to cyclic hypoxia, there was also a depletion of pyruvate concomitant with a slight decrease in oxaloacetate and succinate. The latter is probably accumulated during the hypoxic phase (Chinopoulos, 2019) but is then completely consumed following reoxygenation. This disturbance in energy metabolism could potentially explain the slight slowdown in growth observed during the first hypoxia cycles. Nevertheless, the main concern is that the presence of an additional stressor may not be tolerated by fish whose metabolism is already disturbed during acute cyclic hypoxia.

#### Acclimation

After five hypoxia cycles (D5), glutamate, alanine and BCAA levels displayed the same trend as the first day, indicating a relatively similar metabolic response compared to the first day. The slight increase in pyruvate and in the metabolites of the TCA cycle suggest their accumulation during hypoxia but also a lower pressure on energetic metabolism during reoxygenation, in contrast to the acute response. The lower concentration of glycine contrasts with the increase reported in the common carp liver during hypoxia and in the crucian carp liver during anoxia and recovery (Lardon *et al.,* 2013; Dahl *et al.,* 2021). As a glucogenic amino acid, glycine can contribute to pyruvate production. Glycine is also involved with glutamate in glutathione synthesis and ammonia detoxification in common carp, as shown by Hoseini *et al.* (2022). Glycine concentration probably decreased in Arctic char due to the lower capacity of this species to deal with hypoxia compared to common and crucian carps.

After 10 days of cycling hypoxia, BCAAs no longer accumulate which may reflect down-regulation of protein degradation during hypoxia. Alanine still accumulated during acclimation to cyclic hypoxia after 10 days, but glutamate was not decreased anymore. Additionally, concentrations of metabolites of the TCA cycle that were slightly elevated at D5 returned to control concentrations after 10 days of cyclic hypoxia. Interestingly, fumarate and succinate concentrations are lower after 10 days of cyclic hypoxia. When succinate accumulated during hypoxia is oxidized to fumarate during reoxygenation, reactive oxygen species (ROS) are generated (Chouchani *et al.,* 2014). Keeping the concentration of succinate low during hypoxia may be a strategy to reduce oxidative stress during cyclic hypoxia. This mechanism is even more obvious after 1 month of cyclic hypoxia (D30). Both succinate and fumarate concentrations are lower in the hypoxic group and are the only metabolites that differ in the two groups (Fig. 7B). Furthermore, we observed a reduced, albeit not significant, succinate:fumarate ratio in fish exposed to cyclic hypoxia (Supplementary Fig. 3A). Keeping this ratio low may be viewed as a protective mechanism against ROS. Hypoxia-tolerant species tend to have a lower succinate:fumarate ratio during hypoxia exposure (Bundgaard *et al.,* 2019; Shekhovtsov *et al.,* 2020). Finally, a slightly higher pyruvate:lactate ratio observed in the cycling fish (Supplementary Fig. 3B) indicates a decreased dependence on anaerobic glycolysis.

In general, after 30 days of cycling hypoxia, both groups of fish showed a similar metabolome, suggesting that Arctic char exposed to 1 month of cyclic hypoxia displayed a high metabolic resilience and successfully adopted a phenotype that is more tolerant to cyclic hypoxia.

### Implication for southern Arctic char populations

This study reveals that Arctic char have the ability to acclimate to cyclic hypoxia through efficient phenotypic plasticity. Despite the small decrease of growth observed in the first few days, Arctic char can quickly recover while defending their energy stores. The first few hypoxia events caused profound hepatic metabolome alterations but after 1 month of cyclic hypoxia, most of these effects were mitigated. This supports the transition to a more hypoxia-tolerant phenotype, as was observed in Williams *et al.* (2019). Nevertheless, fish require several days to make physiological adjustments. During this period, the fish’s ability to cope with an additional stressor, such as a heat wave, may be significantly diminished. The rise of cyclic hypoxia episods may therefore exacerbates the decline of the distribution area of Arctic char, already affected by rising temperatures and competition with other species (Hein *et al.,* 2012). Multi-stressor studies would be needed to verify this possibility or to confirm Arctic char’s resilience to cyclic hypoxia in its changing environment. It is worth noting that fish used in this study are from a Fraser strain maintained in an aquaculture facility. Previous studies demonstrated that strain and domestication could induce a change in hypoxia tolerance partly due to maintenance conditions (Gamperl and Farrell, 2004; Scott *et al.,* 2014; Zhang *et al.,* 2016). However, a study carried on Eurasian perch (*Perca fluviatilis*) showed that domestication had a limited impact on physiological response to hypoxia and repeated hypoxia (Douxfils *et al.,* 2012). Therefore, some wild Arctic char populations may have a different critical threshold to cyclic hypoxia, but we expect them to show a similar response to this environmental stressor. By investigating short- and long-term effects of cyclic hypoxia on growth and metabolic processes, this study provides crucial information on how salmonid may cope with the deoxygenation of their habitats. Although numerous research projects focused on the effects of hypoxia, our study is one of the few to consider the cyclic aspect of this stressor which is paramount to comprehending whether and how sensitive fish populations will survive considering the ongoing climate change and environmental degradation.

## Supplementary Material

Web_Material_coad099

## Data Availability

Data are available through the Open Science Framework: DOI 10.17605/OSF.IO/EDXRH.
