# Supplementary material for: Resilience and phenotypic plasticity of Arctic char (Salvelinus alpinus) facing cyclic hypoxia: insights into growth, energy stores and hepatic metabolism
Source: Conserv Physiol. 2023 Dec 15;11(1):coad099. doi: 10.1093/conphys/coad099 (PMC10724465; doi:10.1093/conphys/coad099)
Supplement: Web_Material_coad099 [file web_material_coad099.pdf]

Supplementary Table 1- Means ±*sem* and two-sample t-test results for growth parameters of Arctic char after 30 days of normoxia or cyclic hypoxia (n=5).

	Treatments		Two-sample t-test		
	Normoxia<br>*Mean ±sem*	Cyclic Hypoxia<br>*Mean ±sem*	t-value	df	p-value
**SGR in length**[T]	0.108 ±*0.010 %.day*$^{-1}$	0.113 ±*0.019 %.day*$^{-1}$	-0.006	8	0.996
**SGR in mass**	0.277 ±*0.046 %.day*$^{-1}$	0.323 ±*0. 0.046 %.day*$^{-1}$	-0.705	8	0.501
**CF variation**	-0.045 ±*0.034 %.day*$^{-1}$	-0.014 ±*0.013 %.day*$^{-1}$	-0.862	8	0.414

[T] data log transformation applied for t-test

Supplementary Table 2 – *Post-hoc* statistic test results for SGR in mass, CF variation and hepatic pyruvate:lactate ratio of Arctic char exposed to normoxia or cyclic hypoxia during 30 days (n=5).

	t-ratio	df	p-value
**SGR in mass**			
Normoxia D0-D10 - Cyclic hypoxia D0-D10	1.601	8	0.6195
Normoxia D0-D10 - Normoxia D10-D20	-1.068	16	0.8868
Normoxia D0-D10 - Cyclic hypoxia D10-D20	-3.77	8	0.0429
Normoxia D0-D10 - Normoxia D20-D30	-2.404	16	0.2122
Normoxia D0-D10 - Cyclic hypoxia D20-D30	-2.427	8	0.251
Cyclic hypoxia D0-D10 - Normoxia D10-D20	-2.427	8	0.251
Cyclic hypoxia D0-D10 - Cyclic hypoxia D10-D20	-6.945	16	<.0001
Cyclic hypoxia D0-D10 - Normoxia D20-D30	-3.46	8	0.0647
Cyclic hypoxia D0-D10 - Cyclic hypoxia D20-D30	-5.209	16	0.001
Normoxia D10-D20 - Cyclic hypoxia D10-D20	-2.943	8	0.1287
Normoxia D10-D20 - Normoxia D20-D30	-1.336	16	0.7621
Normoxia D10-D20 - Cyclic hypoxia D20-D30	-1.601	8	0.6195
Cyclic hypoxia D10-D20 - Normoxia D20-D30	1.911	8	0.4585
Cyclic hypoxia D10-D20 - Cyclic hypoxia D20-D30	1.736	16	0.5294
Normoxia D20-D30 - Cyclic hypoxia D20-D30	-0.568	8	0.9906
**CF variation**			
Normoxia D0-D10 - Cyclic hypoxia D0-D10	1.553	8	0.6452
Normoxia D0-D10 - Normoxia D10-D20	-0.336	16	0.9993
Normoxia D0-D10 - Cyclic hypoxia D10-D20	-4.196	8	0.0246
Normoxia D0-D10 - Normoxia D20-D30	-3.763	16	0.0175
Normoxia D0-D10 - Cyclic hypoxia D20-D30	-2.969	8	0.1244
Cyclic hypoxia D0-D10 - Normoxia D10-D20	-1.88	8	0.4733
Cyclic hypoxia D0-D10 - Cyclic hypoxia D10-D20	-5.915	16	0.0003
Cyclic hypoxia D0-D10 - Normoxia D20-D30	-5.211	8	0.0071
Cyclic hypoxia D0-D10 - Cyclic hypoxia D20-D30	-4.652	16	0.003
Normoxia D10-D20 - Cyclic hypoxia D10-D20	-3.869	8	0.0376
Normoxia D10-D20 - Normoxia D20-D30	-3.426	16	0.0338
Normoxia D10-D20 - Cyclic hypoxia D20-D30	-2.642	8	0.191
Cyclic hypoxia D10-D20 - Normoxia D20-D30	0.539	8	0.9926
Cyclic hypoxia D10-D20 - Cyclic hypoxia D20-D30	1.263	16	0.8001
Normoxia D20-D30 - Cyclic hypoxia D20-D30	0.688	8	0.9783
**pyruvate:lactate ratio**			
Normoxia D1 - Cyclic hypoxia D1	3.326	8	0.112
Normoxia D1 - Normoxia D5	1.309	23	0.8862
Normoxia D1 - Cyclic hypoxia D5	2.908	8	0.1902
Normoxia D1 - Normoxia D10	1.126	23	0.9441
Normoxia D1 - Cyclic hypoxia D10	1.844	8	0.6135
Normoxia D1 - Normoxia D30	3.141	23	0.0735
Normoxia D1 - Cyclic hypoxia D30	0.825	8	0.9859
Cyclic hypoxia D1 - Normoxia D5	-2.017	8	0.5238
Cyclic hypoxia D1 - Cyclic hypoxia D5	-0.418	23	0.9999
Cyclic hypoxia D1 - Normoxia D10	-2.2	8	0.4354

Cyclic hypoxia D1 - Cyclic hypoxia D10	-1.292	23	0.8927
Cyclic hypoxia D1 - Normoxia D30	-0.185	8	1
Cyclic hypoxia D1 - Cyclic hypoxia D30	-2.5	23	0.2441
Normoxia D5 - Cyclic hypoxia D5	1.599	8	0.7415
Normoxia D5 - Normoxia D10	-0.183	23	1
Normoxia D5 - Cyclic hypoxia D10	0.61	8	0.9976
Normoxia D5 - Normoxia D30	1.832	23	0.6065
Normoxia D5 - Cyclic hypoxia D30	-0.484	8	0.9994
Cyclic hypoxia D5 - Normoxia D10	-1.782	8	0.6463
Cyclic hypoxia D5 - Cyclic hypoxia D10	-0.898	23	0.9833
Cyclic hypoxia D5 - Normoxia D30	0.233	8	1
Cyclic hypoxia D5 - Cyclic hypoxia D30	-2.082	23	0.4538
Normoxia D10 - Cyclic hypoxia D10	0.782	8	0.9896
Normoxia D10 - Normoxia D30	2.015	23	0.4941
Normoxia D10 - Cyclic hypoxia D30	-0.301	8	1
Cyclic hypoxia D10 - Normoxia D30	1.118	8	0.9352
Cyclic hypoxia D10 - Cyclic hypoxia D30	-1.065	23	0.9578
Normoxia D30 - Cyclic hypoxia D30	-2.315	8	0.3842

Supplementary Table 3 – List of identified metabolites included in the metabolomic analyses.

Metabolites	HMDB ID	Metabolites	HMDB ID
Acetate	HMDB0000042	Glycolate	HMDB0000115
Acetoacetate	HMDB0000060	Isocitrate	HMDB0000193
Adenine	HMDB0000034	Isoleucine	HMDB0000172
Alanine	HMDB0000161	Lactate	HMDB0000190
Argininosuccinate	HMDB0000052	Leucine	HMDB0000687
L-Asparagine	HMDB0000168	Lysine	HMDB0000182
D-Aspartate	HMDB0006483	Malate	HMDB0000744
Beta-Alanine	HMDB0000056	D-Mannose	HMDB0000169
Beta-Leucine	HMDB0003640	Ornithine	HMDB0000214
Carnitine	HMDB0000062	Oxalacetate	HMDB0000223
Citrate	HMDB0000094	Phenylalanine	HMDB0000159
Citrulline	HMDB0000904	Phosphoenolpyruvate	HMDB0000263
Coenzyme A	HMDB0001423	3-Phosphoglycerate	HMDB0000807
Creatine	HMDB0000064	L-Proline	HMDB0000162
L-Cysteine	HMDB0000574	Propionate	HMDB0000237
Fumarate	HMDB0000134	Pyruvate	HMDB0000243
Glucosamine-6-phosphate	HMDB0001254	Serine	HMDB0062263
Glucose	HMDB0000122	Succinate	HMDB0000254
Glucose-6-phosphate	HMDB0001401	Sucrose	HMDB0000258
Glutamate	HMDB0000148	Taurine	HMDB0000251
Glyceraldehyde	HMDB0001051	Threonine	HMDB0000167
Glycerol	HMDB0000131	Tyrosine	HMDB0000158
Glycerol-3-phosphate	HMDB0000126	UDP-glucose	HMDB0000286
Glycine	HMDB0000123	Valine	HMDB0000883
Glycogen	HMDB0000757		

Supplementary Table 4 - Diagnostic statistic values obtained after cross validation (LOOCV method) of PLS-DA computed for normoxia and cyclic hypoxia treatments and for each sampling days.

	**1 component**			**2 components**		
	Accuracy	R2	Q2	Accuracy	R2	Q2
**Treatment**						
Normoxia	0.10	0.80	0.20	0.35	0.94	0.30
Cyclic hypoxia	0.37	0.83	0.57	0.63	0.95	0.59
**Sampling day**						
Day 1	1.00	0.97	0.74	1.00	0.99	0.80
Day 5	0.70	0.78	0.28	0.80	0.94	0.32
Day 10	0.67	0.94	0.21	0.78	0.99	0.26
Day 30	0.40	0.88	-0.21	0.6	0.97	-0.01

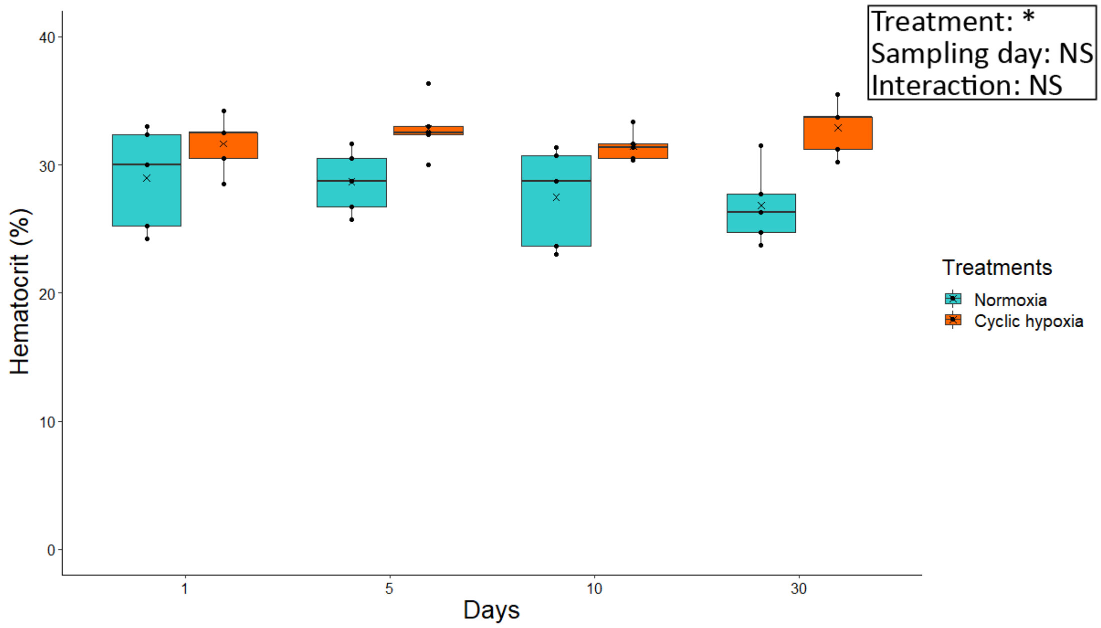

Supplementary Figure 1- Hematocrit of Arctic char at days 1, 5, 10 and 30 after fish were either maintained in normoxia or exposed to cyclic hypoxia (n=5). Box plots indicate the median (—), the mean (×), 25th and 75th percentiles (box), 95% range (|) and each observation (●). Two-way ANOVA results are shown as NS (non-significative), * (p-value<0.05).

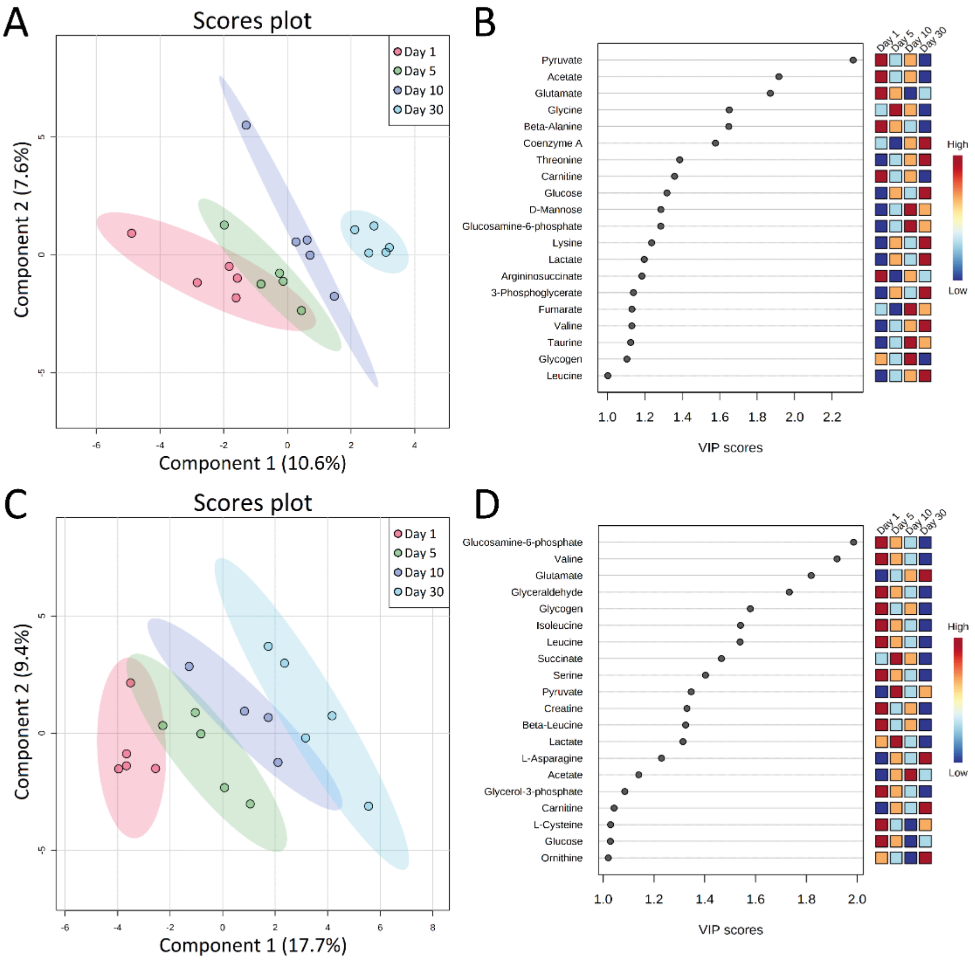

Supplementary Figure 2- Metabolite profile in liver of Arctic char at days 1 (red), 5 (green), 10 (blue) and 30 (cyan) after fish were either maintained in normoxia (A-B) or exposed to cyclic hypoxia (C-D) (n=4-5): A-C) PLS-DA 2D score plots of liver metabolites. Components 1 and 2 represent variance proportion and ellipses correspond to 95% confidence intervals for each sampling day group. B-D) Variable importance in projection (VIP) scores of PLS-DA component 1 for liver metabolites which drive metabolic profile differentiation between sampling days (VIP score >1). Relative concentrations of corresponding metabolite are indicated by colored boxes on the right.

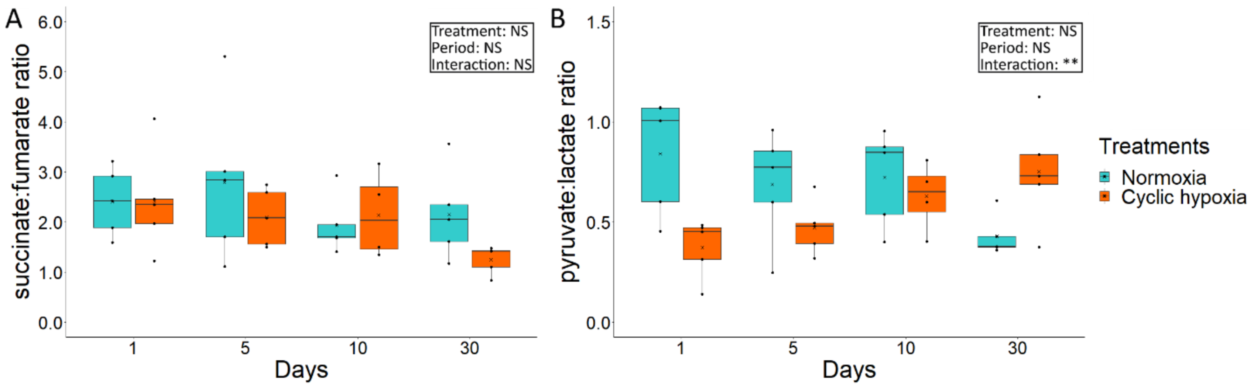

Supplementary Figure 3- Liver metabolite ratios of Arctic char at days 1, 5, 10 and 30 after fish were either maintained in normoxia or exposed to cyclic hypoxia (n=4-5). A) succinate:fumarate ratio. B) pyruvate:lactate ratio. Box plots indicate the median (—), the mean (×), 25th and 75th percentiles (box), 95% range (|) and each observation (●). Two-way ANOVA results are shown as NS (non-significative), ** (p-value<0.01).
